# The potential of exogenous specialized pro-resolving mediators in protecting against sepsis-associated lung injury: a review

**DOI:** 10.3389/fphar.2025.1622754

**Published:** 2025-07-29

**Authors:** Jiwei Shen, Huiqin Shen, Shujun Sun, Shiwen Fan, Tianhao Zhang, Guobin Song, Ning An, Xiangdong Chen, Yafen Gao

**Affiliations:** ^1^Department of Anesthesiology, Union Hospital, Tongji Medical College, Huazhong University of Science and Technology, Wuhan, China; ^2^Institute of Anesthesia and Critical Care Medicine, Union Hospital, Tongji Medical College, Huazhong University of Science and Technology, Wuhan, China; ^3^Key Laboratory of Anesthesiology and Resuscitation (Huazhong University of Science and Technology), Ministry of Education, Wuhan, China

**Keywords:** specialized pro-resolving mediators (SPMs), sepsis-associated lung injury, lipoxin, resolvin, protectin, maresins

## Abstract

Sepsis-associated lung injury (SALI) is a critical condition with high mortality. Current therapies are limited, necessitating novel approaches. This review highlights the potential of exogenous Specialized Pro-resolving Mediators (SPMs), including lipoxins, resolvins, protectins, and maresins, in mitigating SALI. SPMs, derived from polyunsaturated fatty acids, exert protective effects through multiple mechanisms: enhancing alveolar fluid clearance by upregulating ENaC, Na,K-ATPase, CFTR, and AQP5; reducing alveolar epithelial cell apoptosis and epithelial-mesenchymal transition; preserving endothelial glycocalyx integrity via modulating heparanase and exostosin-1 expression; alleviating oxidative stress and mitochondrial dysfunction by scavenging ROS and activating Nrf2; and immunomodulation by limiting neutrophil infiltration, promoting macrophage efferocytosis and M2 polarization, and dampening pro-inflammatory cytokine production. Notably, SPMs like RvD2 remain effective even during sepsis’ immunosuppressive phase. While significant debates persist regarding endogenous SPM generation, receptor mechanisms, and critically the reliable detection and physiological relevance of specific SPMs in biological samples like lung tissue (with earlier reports often misidentifying analytical artifacts or failing LOD/LOQ validation), recent evidence suggests exogenous SPMs act via biased allosteric modulation of the EP4 receptor to stimulate phagocytosis and resolution. Extensive preclinical evidence underscores SPMs’ promise in restoring immune homeostasis in SALI, though pharmacokinetic limitations of high-dose exogenous administration require consideration. Future high-quality clinical trials are essential to translate this resolution pharmacology approach into clinical practice.

## 1 Introduction

Sepsis is a life-threatening organ dysfunction syndrome due to dysregulation of the body’s response to infection. It is a major risk factor for acute lung injury (ALI) and acute respiratory distress syndrome (ARDS), accounting for 31% of the etiology (Bersten et al., 2002). The mortality of sepsis-induced ALI/ARDS is significantly higher than that induced by non-sepsis factors. Moreover, phenotype for sepsis that patients had more inflammation and pulmonary dysfunction have relatively higher 28-day mortality compared to some other phenotypes (Seymour et al., 2019). The pathophysiological mechanisms involved in sepsis-associated lung injury (SALI) include uncontrolled inflammatory immunity, endothelial-alveolar epithelial barrier damage, pulmonary oedema and respiratory failure. Currently, supportive therapies such as anti-infective, lung protective ventilation and fluid management strategies remain the mainstay of clinical management of SALI, which, despite significant advances over the past few years, remains a serious clinical problem with a high mortality rate (Xu et al., 2023). It is imperative to explore new strategies for modulating inflammatory-immune disorders in order to improve the prognosis of patients with SALI.

The resolution of inflammation is increasingly recognized as an active and potentially modulatable process. A substantial body of experimental research demonstrates that exogenously administered specialized pro-resolving mediators (SPMs) can promote pro-resolving effects in preclinical models (Serhan, 2017; Fredman et al., 2016; Chattopadhyay et al., 2017; Wang L. et al., 2014). SPMs, a category encompassing lipoxins (LX), resolvins (Rv), protectins (PD), and maresins (MaR) based on their proposed structural classifications, and this category also includes conjugated tissue regenerators (CTRs), proposed to form during tissue regeneration (Serhan et al., 2008; Fredman and Serhan, 2024). Critically, the physiological relevance of endogenous SPM generation, their typical *in vivo* concentrations, and their specific receptor interactions remain active topics of scientific debate and uncertainty. Significant analytical controversies exist regarding reliable detection of SPMs in biological samples. Numerous publications have erroneously presented chromatographic artifacts (e.g., solvent peaks, contaminants) as evidence for SPMs like RvD2 or RvE1 in sepsis or lung injury contexts, often due to failures in applying standard limit-of-detection (LOD) or limit-of-quantitation (LOQ) criteria (O'Donnell et al., 2023). Consequently, internationally agreed technical standards for oxylipin analysis by LC-MS/MS have now been established to ensure rigorous identification and quantification (Schebb et al., 2025). Notably, no study to date has definitively demonstrated the presence of SPMs (e.g., RvD2, RvE1) in SALI samples using validated methods adhering to these standards. Despite these unresolved questions regarding endogenous biology, the exogenous application of certain SPMs has shown promise in models. Unlike broad immunosuppressive agents, exogenously administered SPMs are proposed to offer a potential therapeutic advantage for conditions like sepsis by reportedly stimulating the resolution of inflammation without globally compromising host defense mechanisms, thereby promoting a return to homeostasis (Buckley et al., 2014). Numerous studies have investigated the protective effects of exogenously administered SPMs in experimental SALI (Chattopadhyay et al., 2017; Jin et al., 2007; El Kebir et al., 2009). However, a systematic review comprehensively summarizing these findings, their underlying mechanisms, and the associated scientific controversies is lacking. This article aims to review the reported protective roles of exogenously administered SPMs in SALI, examining perspectives including lung epithelial and endothelial injury, oxidative stress and mitochondria-related mechanisms, and immunology, while critically evaluating their therapeutic potential in light of ongoing debates.

## 2 A brief overview of the biological role of SPMs in inflammation-resolution

Traditional understanding posits that SPMs are biosynthesized endogenously from polyunsaturated fatty acids (PUFAs) like arachidonic acid (AA), eicosapentaenoic acid (EPA), and docosahexaenoic acid (DHA). This process is described as involving key enzymes such as cyclooxygenase-2 (COX-2), particularly in its aspirin-acetylated form, arachidonate 5-lipoxygenase (5-LOX), and 12/15-LOX. Multiple cell types, including leukocytes, platelets, epithelial cells, and endothelial cells, have been implicated in this biosynthesis, potentially occurring within single cells or through transcellular pathways (Serhan and Petasis, 2011; Basil and Levy, 2016). Furthermore, specific G protein-coupled receptors (GPCRs) such as ALX/FPR2 for lipoxins (e.g., LXA4), ChemR23 and BLT1 for RvE1, GPR32/ALX for RvD1, GPR18 for RvD2, GPR37 for PD1, and LGR6 for maresin 1 (MaR1) have been proposed as mediators of SPM actions (Dyall et al., 2022; Jordan and Werz, 2022; Serhan and Chiang, 2023; Serhan et al., 2022; Serhan and Levy, 2018; Basil and Levy, 2016). Based on this framework, SPMs were suggested to orchestrate inflammation resolution by modulating immune cell functions: reducing neutrophil recruitment, adhesion, and activation (Gong et al., 2014); promoting neutrophil apoptosis and macrophage efferocytosis/phagocytosis; shifting macrophage polarization towards an M2 phenotype (Godson et al., 2000); dampening dendritic cell pro-inflammatory cytokine production (Haworth et al., 2008); enhancing natural killer (NK) cell cytotoxicity (Barnig et al., 2013); regulating innate lymphoid cell type 2 (ILC2) responses (Krishnamoorthy et al., 2015); and influencing B and T cell differentiation, particularly promoting regulatory T cell (Treg) activity (Ramon et al., 2012; Chiurchiù et al., 2016; Oner et al., 2021).

However, significant controversies surround the endogenous formation, physiological relevance, and receptor specificity of SPMs. Critiques highlight challenges in reliably detecting endogenous SPMs at physiologically relevant concentrations using liquid chromatography-tandem mass spectrometry (LC-MS/MS). Concerns have been raised about the validity of data in numerous publications, including reports of unusual chromatographic features and methodological issues. Studies investigating the aspirin-triggered pathway specifically found that while acetylated COX-2 inhibits prostanoids, generation of significant amounts of 15-epi-LXA4 was undetectable in cell models even under high substrate (AA) and supratherapeutic aspirin conditions (Hofling et al., 2022). Furthermore, human studies have largely failed to demonstrate consistent increases in SPM levels in response to dietary ω-3 PUFA supplementation or during the resolution phase of evoked inflammation (Schebb et al., 2022). The proposed specific SPM receptors have also been questioned, with recent high-profile evidence suggesting that several SPMs (e.g., protectins, maresins, D-series resolvins) may function primarily as biased positive allosteric modulators of the prostaglandin E2(PGE2) receptor EP4 at concentrations higher than those typically detected endogenously, enhancing cAMP signaling and phagocytosis via EP4 rather than acting through their originally proposed cognate GPCRs. In the absence of EP4, these SPMs lose their activity (Alnouri et al., 2024).

Despite these ongoing debates regarding endogenous generation and specific receptor mechanisms, a substantial body of preclinical research utilizing exogenous administration of synthetic SPMs demonstrates compelling biological effects relevant to inflammation resolution. Numerous studies consistently report that adding defined SPMs *in vitro* or administering them *in vivo* in various disease models, including sepsis, elicits potent anti-inflammatory and pro-resolving activities (Hu et al., 2020; Tan et al., 2018; Yang et al., 2013; Li et al., 2014; Fredman and Serhan, 2024). These effects manifest as reduced neutrophil infiltration and activation, enhanced macrophage phagocytosis and efferocytosis, modulation of macrophage polarization, suppression of pro-inflammatory cytokine (e.g., TNF-ɑ, IL-1β, IL-6) and chemokine production, promotion of neutrophil apoptosis, and regulation of lymphocyte responses. The mechanisms underlying these observed effects of exogenous SPMs, explored in detail in the subsequent sections of this review, offer significant therapeutic promise, irrespective of the resolution of the controversies surrounding their precise endogenous origins and signaling.

## 3 Protective effects of exogenous SPMs in SALI toward lung epithelial cells

### 3.1 Improve alveolar fluid clearance (AFC)

Alveolar epithelial cells (AECs) are composed of type I (AT I) and type II (AT II) cells. In addition to gas exchange, AFC is also a major function of AECs. The pathologic features of SALI mainly include impaired vascular capillary barrier and impaired clearance of edema fluid, and the ability to clear edema fluid is closely related to the prognosis (Morty et al., 2007). AFC is associated with the active transport of sodium ions in the alveolar epithelium through the apical epithelial sodium channel (ENaC) and basolateral Na,K-ATPase (Eaton et al., 2009; Sznajder et al., 2002). LXA4 could increase the expression of ENaC α and ENaCγ subunit proteins and Na,K-ATPase activity in primary rat AT II cells stimulated with lipopolysaccharide (LPS), thereby activating AFC (Wang et al., 2013). RVD1, PDX, MaR1, MCTR1 and PCTR1 all had similar effects, and the mechanism partially involved the ALX/cAMP/PI3K pathway (Wang Q. et al., 2014; Zhuo X. J. et al., 2018; Zhang et al., 2017; Han et al., 2020; Zhang et al., 2020). Nedd4-2, an E3 ubiquitin protein ligase, is crucial for negative regulation of Na + transport. The above SPMs inhibited the LPS-induced elevation of Nedd4-2 protein expression. Similarly, RvE1 promoted AFC by activating the PI3K/AKT/SGK1 (serum- and glucocorticoid-induced kinase 1) pathway to promote phosphorylation of Nedd4-2 and upregulate the expression of ENaC and Na,K-ATPase (Luo et al., 2022). In addition, PCTR1 promoted the expression of lymphatic vessel endothelial receptor-1 (LYVE-1), restored lymphatic drainage, and led to increasing the clearance of pulmonary interstitial fluid (Zhang et al., 2020). Some researchers had further investigated the mechanism by which LXA4 regulates ENaC-γ in LPS-induced lung injury. In A549 cells, miR-21 upregulated phosphorylation of AKT activation through inhibition of phosphatase and tensin homolog (PTEN), thereby decreasing ENaC-γ expression. Conversely, LXA4 reversed LPS-inhibited ENaC-γ expression through inhibition of activator protein 1 (AP-1), a conserved enhancer element in the miR-21 promoter region, and activation of PTEN (Qi et al., 2015).

In addition to ENaC and Na,K-ATPase, chloride channels and aquaporins (AQPs) also play an important role in the integrity of barrier function during fluid transport. Cystic fibrosis transmembrane conductance regulator (CFTR) is a chloride ion channel expressed in both alveolar AT-I and AT-II cells. Lack or inhibition of CFTR results in a deficient increase in AFC in response to β-adrenergic agonists (Folkesson and Matthay, 2006). LXA4 enhanced CFTR protein expression and increased AFC via the PI3K/Akt pathway (Yang et al., 2013). AQP5 is specifically expressed in the apical membrane of submucosal gland cells and AT-I cells. LPS stimulation was found to increase the permeability of lung epithelial cells by altering the expression and distribution of AQP5 on the cell surface. In the LPS-induced ALI model in rats, LXA4 upregulated AQP5 expression by inhibiting phosphorylation of p38 and JNK, restored AFC function, and exerted a protective effect against SALI (Ba et al., 2019).

**FIGURE 1:**
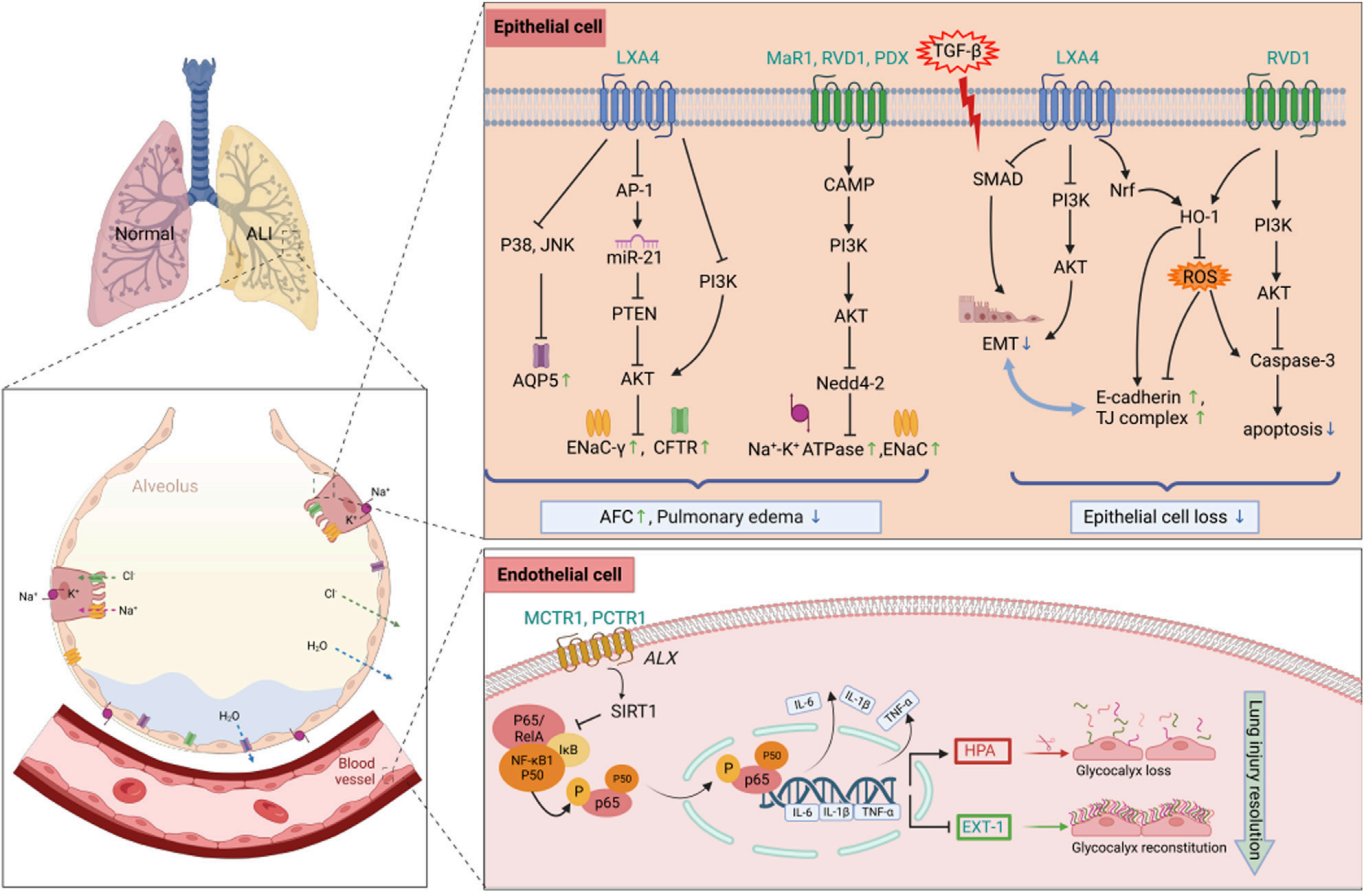
Protective mechanisms of SPMs against epithelial and endothelial cells in septic lung injury. SPMs play a protective role in septic lung injury by restoring the function of AFC to attenuate pulmonary edema, as well as reducing apoptosis and EMT in alveolar epithelial cells, meanwhile, maintaining the homeostasis of glycocalyx through the downregulation of HPA and the upregulation of EXT-1 expression. Abbreviations: SPMs, specialized pro-resolving mediators; AFC, alveolar fluid clearance; EMT, epithelial-mesenchymal transition; HPA, heparanase; EXT-1, exostosin-1; ALI, acute lung injury; AQP5, Aquaporin; TJ complex, tight junction complex.

To summarize, in sepsis, SPMs can ameliorate AFC and attenuate pulmonary edema from multiple targets of ENaC, Na,K-ATPase, CFTR, and AQP5, suggesting their potential application as anti-inflammatory treatment for the impairment of alveolar fluid transport in SALI (Figure 1).

### 3.2 Attenuate AECs injury/loss

In ARDS, the degree of alveolar epithelial destruction is closely related to the prognosis. Extensive apoptosis of AECs is the main result of alveolar epithelial destruction, leading to increased alveolar capillary permeability (Matthay and Zemans, 2011). Repair of alveolar epithelium is necessary for the repair of lung function. Abnormal regulation of repair mechanisms, such as epithelial-mesenchymal transition (EMT), can lead to fibroblast/myofibroblast differentiation, resulting in clinically significant pulmonary fibrosis (Andersson-Sjöland et al., 2008; Omenetti et al., 2008). Thus, apoptosis of AECs and EMT play a crucial role in the development of ARDS. In LPS-induced lung injury, LXA4 promoted the proliferation of AT-II cells, inhibited apoptosis, and decreased the expression of cleaved caspase-3. In addition, LXA4 was found to promote the expression of E-cadherin, an epithelial cell marker, and downregulate the expression of mesenchymal cell markers (including N-cadherin, α-SMA, and vimentin). Mechanistically, LXA4 inhibited EMT, in part through the SMAD and PI3K/AKT signaling pathways in an ALX receptor-dependent manner (Yang et al., 2019). Notably, LXA4 also mediated E-cadherin expression to protect respiratory epithelium from LPS damage by phosphorylating NF-E2-related factor 2 (Nrf2) on Ser40 and triggering its nuclear translocation to activate Nrf2 and reduce reactive oxygen species (ROS) (Cheng et al., 2016). Similarly, RvD1 reduced apoptosis (Xie et al., 2016), promoted epithelial wound repair and inhibited TGF-β-induced EMT, while reducing fiberproliferation and collagen production in primary human alveolar epithelial type 2 cells (Zheng et al., 2018). In addition, RvD1 significantly attenuated the degradation of tight junction (TI) proteins occludin and zona occludin-1 (ZO-1), thereby reducing LPS-induced permeability edema in mice (Figure 1). The heme oxygenase-1(HO-1) inhibitor, zinc protoporphyrin-9 (Znpp IX), reversed this effect, suggesting that HO-1 mediates, at least in part, the lung-protecting effects of RVD1 (Xie et al., 2013). Ocdcludin and ZO-1 are expressed by AECs as part of the TJ complex. TJ proteins establish a regulated paracellular barrier between epithelial and endothelial cells that prevents the movement of water, solutes, and immune cells, and their dysfunction can lead to barrier damage and pulmonary edema in SALI.

In conclusion, these results suggest to us a potentially interesting therapeutic strategy in which SPMs might be used to prevent and treat the fibroproliferative phase of sepsis-induced ARDS. The underlying mechanisms of the antifibrotic effects of SPMs require further experimentation.

## 4 Protective effects of exogenous SPMs in SALI toward lung endothelial cells

Endothelial cells play a regulatory role in the pathophysiology of lung injury. The endothelial glycocalyx layer (EGL) covers the vascular endothelial cells, forming a large vascular endothelial surface layer (ESL), which consists mainly of glycosaminoglycans and proteoglycans, and is degraded to produce heparan sulfate (HS), hyaluronic acid (HA), and syndecan-1 (SDC-1) by the activation of heparanase (HPA) (Chappell et al., 2008). The ESL also inhibits microvascular thrombosis and helps regulate leukocyte adhesion to the endothelium. In sepsis-associated acute lung injury, the breakdown of the ESL results in barrier disruption and leads to lung injury by promoting pulmonary edema and neutrophil adhesion. MCTR1 upregulated SIRT1 expression, decreased NF-κB p65 phosphorylation and downregulated HPA protein expression through the ALX/SIRT1/NF-κB/HPA pathway, which significantly inhibited HS degradation and thus reduced endothelial glycocalyx damage, thereby improving the survival of SALI mice (Li et al., 2020). Exostosin-1 (EXT-1) participates in the synthesis and reconstruction of glycocalyx, but this process is delayed in sepsis. PCTR1 could also upregulate the expression of EXT-1 to promote glycocalyx reconstruction and protect endothelial cells (Wang et al., 2021). Figure 1 summarizes the protective mechanisms of SPMs against lung epithelial and endothelial cells in SALI.

## 5 Reduce oxidative stress and mitochondrial dysfunction

After endotoxins and microorganisms invade lung tissue, large amounts of inflammatory factors released by phagocytic cells and endothelial cells can activate effector cells such as alveolar macrophages and neutrophils, leading to the release of a large number of ROS that damage AECs and vascular endothelial cells, affecting gas exchange in the alveoli and ultimately leading to severe lung injury. Oxidative stress reactions develop in the progression of inflammation, and have a positive feedback effect on the inflammation itself. Oxidative stress and mitochondrial dysfunction are considered to be one of the important pathogenic mechanisms in sepsis (Wen et al., 2015). Direct inhibition of the respiratory chain complex by microbial toxins and pro-inflammatory mediators is thought to be a major factor contributing to the decline in mitochondrial energy production and the initiation of the intrinsic apoptotic pathway in various cells and tissues. In severe inflammation, damaged mitochondria release a number of danger-associated molecular patterns (DAMP), such as ROS, while excessive production of ROS may also lead to structural damage of mitochondrial DNA and proteins, thereby causing mitochondrial dysfunction.

**FIGURE 2:**
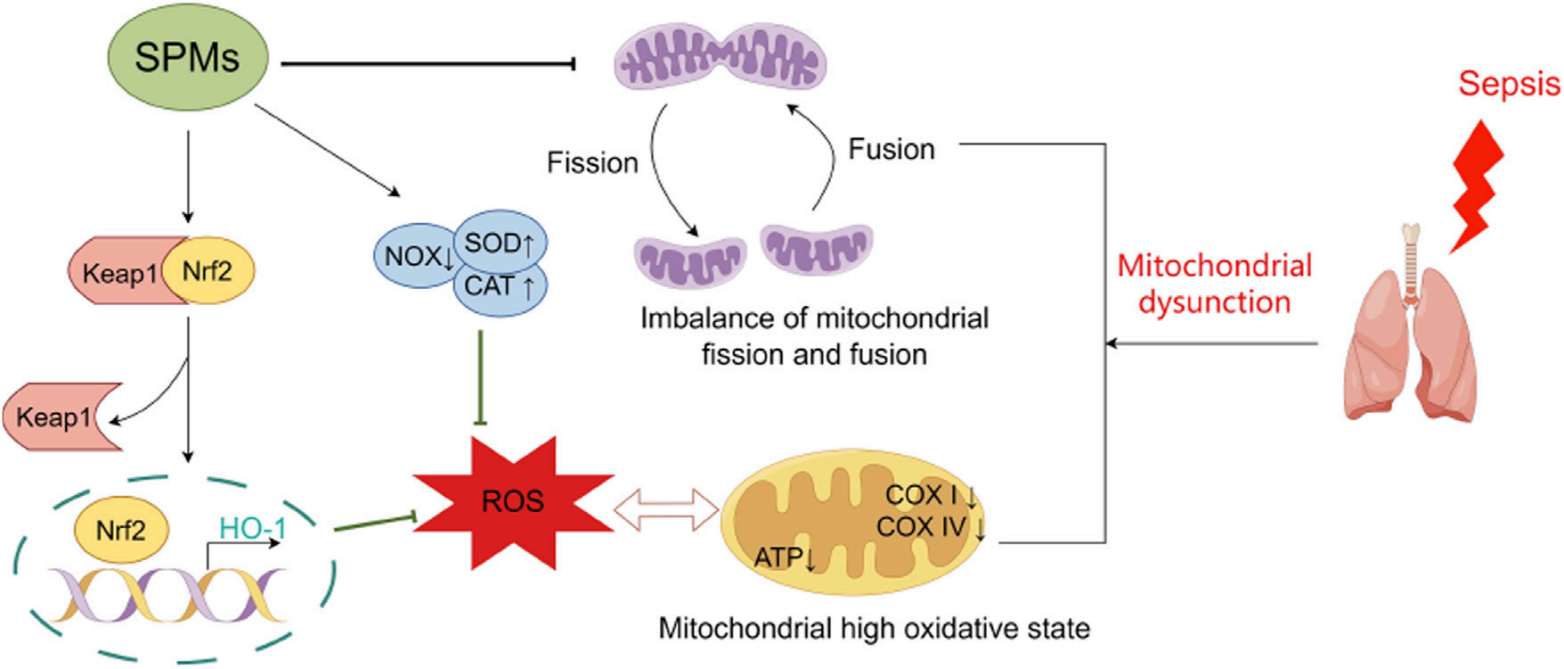
Protective mechanisms of SPMs against oxidative stress and mitochondrial dysfunction in septic lung injury. SPMs play a protective role in septic lung injury by attenuating the impaired mitochondrial respiratory function induced by excess reactive oxygen species, correcting the hyperoxidative state of mitochondria, and ameliorating the imbalance in mitochondrial fission-fusion kinetics. Abbreviations: SPMs, specialized pro-resolving mediators; NOX, NADPH oxidase; CAT, catalase; SOD, superoxide dismutase; ROS, reactive oxygen species.

SPMs have anti-oxidative stress effects and can protect cells from oxidative damage and mitochondrial dysfunction by scavenging free radicals, inhibiting oxidative enzyme activity, and regulating redox homeostasis (Figure 2). Post-treatment of Aspirin-Triggered LXA4 inhibited malondialdehyde (MDA) production and was associated with upregulation of heme oxygenase-1 formation (Jin et al., 2007). Nrf2 is an important transcription factor that regulates the redox state of cells and the production of ROS by modulating phase II detoxifying/antioxidant enzymes. LXA4 activated Nrf2 and triggered its nuclear translocation to attenuate ROS production, thereby reducing LPS-induced acute lung injury in mice (Cheng et al., 2016). Moreover, LXA4 modulated the redox status of mitochondria in the lungs of mice, which were less oxidized in the LXA4 group compared to controls (Cheng et al., 2016). In a cecum ligation and perforation (CLP) mouse model, MaR1 downregulated mitochondrial NADPH oxidase (NOX) activity and increased catalases (CAT) and SOD activities, thereby inhibiting excessive ROS production and attenuating mitochondrial dysfunction in mice, which in consequence attenuated lung injury (Gu et al., 2018). Interestingly, it was found that the effect of MaR1 on ROS generation in the blood of endotoxin-stimulated healthy volunteers or septic patients was associated with ALX and cAMP, which is consistent with animal experiments. Consistent with this, a ^1^H NMR-based metabolomics analysis showed that MaR1 increased lung tissue taurine levels in CLP-induced septic mice, which could enhance the antioxidant defence system by protecting antioxidant enzymes (Hao et al., 2019). In addition, the protective effect of MCTR3 against LPS-induced ALI was partially mediated through the inactivation of the mitophagy pathway mediated by ALX/PTEN-induced putative kinase 1 (PINK1) (Zhuang et al., 2021). A recent study also found that PCTR1 treatment ameliorated mitochondrial ultrastructural damage, dampened ferroptosis through activation of ALX/protein kinase A (PKA)/cAMP-response element binding protein (CREB) and thereby ameliorated LPS-induced ALI (Lv et al., 2023).

Mitochondrial fission and fusion are critical for mitochondrial biogenesis and dynamics, and serve as new perspectives for a better understanding of the mitochondrial stress response. Inflammation-stimulated mitochondrial dysfunction in the human AEC line is reflected in impaired mitochondrial respiration, and altered fission and fusion are detected in these cells. Under TNF-α stimulation, the cellular maximal respiratory capacity was significantly reduced, and the expression of fission-regulating gene FIS1 was up-regulatedand while the fusion-associated gene MFN1 was down-regulatedand. The use of the mitochondrial fission inhibitor Mdivi-1 reduced the concentrations of the pro-inflammatory cytokines IL-6 and IL-8. RvE1 restored the impairment of mitochondrial respiratory capacity and imbalance of mitochondrial fission and fusion induced by inflammation, suggesting a novel functional mechanism for the beneficial role of RvE1 in pulmonary inflammatory responses (Mayer et al., 2019).

## 6 Immunologic perspectives on the protective role of exogenous SPMs in SALI

### 6.1 Immune cells

Circulatory inflammatory overload associated with sepsis leads to endothelial damage to pulmonary capillaries and inflammatory lung injury, which involves a variety of immune cells. As we mentioned before, SPMs can act on a variety of innate and adaptive immune cells to achieve its pro-resolving effects. The biological mechanisms behind the protective effects of SPM-targeted immune cells in SALI are beginning to be elucidated, focusing primarily on neutrophils and macrophages.

Activation of alveolar space neutrophils is a common feature of ALI in humans and animal models. During septic episodes, circulating neutrophils undergo defomation, adherence, aggregation, migration, and infiltration into lung tissue, and their own secretion of cytotoxic products and neutrophil extracellular traps (NETs) production can also lead to alveolar epithelial and endothelial damage. PD1 protects against LPS-induced ALI by inhibiting neutrophil infiltration and NETs formation in lung tissue (Wu et al., 2021). Similarly, the newly identified 13-series (T-series) resolvins (RvTs) reduced NETosis and enhanced NETs clearance by macrophages through a cyclic adenosine monophosphate/PKA/AMPK axis (Chiang et al., 2022). MaR1 reduced the expression of intercellular a dhesion molecule (ICAM)-1, P-selectin and CD24, and attenuated LPS-induced lung injury by inhibiting neutrophil adhesion and the production of pro-inflammatory mediators (Gong et al., 2014). When the vasculature is damaged, endothelial cells release a range of signalling molecules to attract surrounding neutrophils, while platelets aggregate at the site of damage and interact with endothelial cells and neutrophils, further enhancing neutrophil activation and recruitment. AT-15-Epi-LXA4 and PDX have also been shown to alleviate ALI in mice by regulating neutrophil-platelet aggregation (Ortiz-Muñoz et al., 2014; Tan et al., 2018). RvD1 could regulate bronchoalveolar lavage fluid (BALF) neutrophils accumulation through CXCL-12/CXCR4 pathway (Yaxin et al., 2014). Lung tissue expressed more CXCL-12, also known as stromal cell derived factor 1 (SDF-1),in ALI (Petty et al., 2007) and CXCR4 plays an important role in homing of aged neutrophils to bone marrow for clearance (Allen et al., 2004). RvD1 reduced the expression of CXCL-12 mRNA in lung tissue, and promoted the expression of CXCR4 in neutrophils in the early stage of inflammation (24 h) while decreased the expression of CXCR4 in the later stage of inflammation (72 h), thereby regulating neutrophil aggregation in BALF and alleviating LPS-induced ALI (Yaxin et al., 2014). Apoptosis is considered the main mechanism for clearing neutrophils from the site of inflammation (Moon et al., 2010). In sepsis, neutrophil apoptosis is suppressed, and the apoptosis process of neutrophils becomes one of the control points for the resolution of inflammation (Gong et al., 2015). Myeloperoxidase (MPO) is expressed in large quantities in neutrophils, not only producing cytotoxic oxidants but also signaling through β2 integrin CD11b/CD18 (Mac-1) to rescue neutrophils from apoptosis, thereby prolonging inflammation (El Kebir et al., 2008). In an *E. coli* SALI model, 15-Epi-LXA4 promoted neutrophil caspase-3-mediated apoptosis by inhibiting MPO-induced extracellular signal-regulated kinase (ERK) and Akt-mediated phosphorylation of the pro-apoptotic protein Bad and reducing expression of the anti-apoptotic protein Mcl-1 (El Kebir et al., 2009). Notably, Wang et al. applied single-cell sequencing to reveal, at the single-cell level, that maresin1 significantly reduced neutrophil infiltration in SALI, and that this regulatory effect was more focused in the neutrophil-Cxcl3 subpopulation (Wang et al., 2022).

Inflammatory lungs contain two groups of alveolar macrophages: resident alveolar macrophages (RAMs) and recruited macrophages. During the acute inflammatory phase of SALI, RAMs are immediately activated and switched to M1 macrophages, which release cytokines and chemokines to recruit neutrophils, monocytes, *etc.*, to trigger lung inflammation (Arango Duque and Descoteaux, 2014). However, in the late rehabilitation phase, both resident and recruited macrophages shift to M2 phenotype, which exert anti-inflammatory and pro-resolving effects mainly through phagocytosis and efferocytosis to avoid excessive inflammatory damage (Herold et al., 2011). Macrophages possess a unique role and amazing adaptability that renders them a promising target for the management of SALI. CXCL2, also known as macrophage inflammatory protein 2 (MIP-2), mainly functions to induce neutrophil chemotaxis to inflammatory tissues. Monocyte chemoattractant protein-1 (MCP-1) is a widely expressed monocytes and macrophages chemokine. It was found that in lungs, MIP-2 and MCP-1 were predominantly present on RAMs (Ye et al., 2020). In LPS-induced ALI, RvD1 inhibited MIP-2 expression on RAMs, thereby suppressing neutrophil infiltration (Zhang H. W. et al., 2019). Similarly, PDX attenuated LPS-induced lung injury by inhibiting neutrophil and recruited macrophage recruitment through suppression of MIP-2 and MCP-1 expression, respectively (Ye et al., 2020). In this study, it was also suggested that tumor necrosis factor-ɑ (TNF-ɑ) was expressed mainly on recruited macrophages, and that the mechanism by which PDX inhibited neutrophil infiltration and transmembranes may be related to TNF-ɑ/MIP-2/MMP-9 signaling pathway (Ye et al., 2020). Thus, both RAMs and recruited macrophages serve as targets for PDX to reduce inflammatory cell infiltration. Moreover, in a CLP model, PDX was able to promote M2 polarization of peritoneal macrophages and enhance their phagocytic activity to reduce the bacterial load and accelerate the resolution of inflammation, thus improving the survival of septic mice and reducing multiple organ damage, including lungs (Xia et al., 2017). Also in the LPS-induced lung injury model, Mar1 was shown to promote M2 macrophage polarization and accelerate the resolution of ALI by a mechanism related to activation of PPAR-γ(Qiao et al., 2020). Another study explored the effects of MCTR1 on M2 polarization of resident and recruited macrophages and found that MCTR1 enhanced inflammatory resolution and attenuated LPS-induced lung injury primarily by promoting resident M2 macrophage polarization, which was mediated through the STAT6 pathway (Wang et al., 2020). A recent study also found that RvD1 promoted RAMs self-renewal and phagocytosis through the ALX/MAPK14/S100A8/A9 signaling pathway in a mouse model of aerosolized inhaled LPS and/or *E. coli* (Ye et al., 2025). Notably, autophagy is also one of the mechanisms by which SPMs target and regulate macrophage function. In a study using BML-111, an LXA4 receptor agonist, the authors found that BML-111 targeted the MAPK pathway to stimulate autophagy in AMs, significantly reducing LPS-induced AMs apoptosis, thereby suppressing inflammation and ameliorating lung injury (Liu et al., 2018).

### 6.2 Inflammatory cytokines and inflammatory signal pathways

Several SPMs have been shown to reduce LPS-induced pro-inflammatory cytokines (such as TNF-ɑ, IL-1β, and IL-6), chemokines (such as keratinocyte-derived chemokine, MCP-1, and MIP-1), thereby attenuating LPS-induced ALI and improving survival in septic mice (Gong et al., 2014; Jin et al., 2007; Walker et al., 2011; Wang et al., 2011; Xia et al., 2020). Signaling pathways involved are related to NF-κB, signal transducer and activator of transcription (STAT), MAPKs (Wang et al., 2011; Hu et al., 2020; de Oliveira et al., 2017; Li et al., 2016). It is well known that NF-κB, MAPKs, STAT are important transcription factors that regulate the expression of inflammatory genes and are thought to be involved in the pathogenesis of sepsis. The MAPKs pathway has three major subfamilies, including the ERK cascade, c-Jun NH2-terminal/stress-activated protein kinase (JNK/SAPK) cascade, and p38-MAPK cascade. In a CLP septic mouse model, RvD1 promoted the resolution of inflammation by upregulating SIRT1 expression and inhibiting the activation of STAT3, ERK, p38, and NF-κB in lung tissue, thereby reducing the severity of pulmonary inflammation (Zhuo Y. et al., 2018). In an *in vitro* experiment using LPS-stimulated human bronchial epithelial cells (BEAS-2B), AT-RvD1 reduced the concentration of the chemokine CCL-2 in dependence on FPR2/ALX, mechanistically related to its downregulation of the phosphorylation of NF-κB, STAT1 and STAT6 (de Oliveira et al., 2017). In a mouse model of *E. coli* pneumonia, 15-epi-LXA4-ALX/FPR2 interaction selectively upregulated the NF-κB negative regulators A20 and SIGIRR, thereby decreasing NF-κB activity, which may inhibit pulmonary neutrophil infiltration while increasing bacterial clearance and improving sepsis survival (Sham et al., 2018). Furthermore, RvD1-and PDX-induced IκBα degradation and p65 nuclear translocation inhibition were dependent on PPARγ (Liao et al., 2012; Xia et al., 2020).

Of note is the dual effect of LXA4 on LPS-stimulated COX2 expression in lung fibroblasts (Zheng et al., 2011). Exogenous LXA4 inhibited the first peak of LPS-induced COX-2 expression as well as PGE2 production in a dose-dependent manner. In contrast, LXA4 increased the second peak of LPS-induced COX-2 expression and prostaglandin D2 (PGD2) production in a dose-dependent manner. Notably, PGE2 and PGD2 are responsible for regulating the switch of lipid mediator classes from proinflammation to proresolution (Levy et al., 2001). RvD1 effectively promoted p50 homodimer nuclear translocation and upregulated COX-2 expression. The absence of p50 in knockout mice prevented RvD1 from promoting COX-2 and PGD2 expression, leading to excessive lung inflammation. It suggestd that RvD1 accelerated the reslution of inflammation through the NF-kB p50/p50-cox-2 signaling pathway (Gao et al., 2017).

### 6.3 Effects on microorganisms

SPMs can reduce the bacterial load in mouse serum or lungs, such as *E. coli* (Chiang et al., 2012; Abdulnour et al., 2016), *Streptococcus* pneumoniae (Wang et al., 2017), *Pseudomonas aeruginosa* (Abdulnour et al., 2016), and Nontypeable *Haemophilus* influenzae (Croasdell et al., 2016) by enhancing phagocytosis by macrophages and neutrophils. Remarkably, LXA4 not only regulates the host response, but also influences bacterial virulence and enhances antibiotic efficacy. *Pseudomonas aeruginosa* is a gram-negative, opportunistic bacterium that often attacks immunocompromised patients such as those with sepsis. LXA4 inhibited the quorum sensing receptor LasR, thereby reducing *P. aeruginosa* exotoxin release (Wu et al., 2016). In addition, LxA4 reduced the formation of *P. aeruginosa* biofilm and the expression of virulence genes, and enhanced the inhibitory effect of ciprofloxacin on the formation of biofilm and its bactericidal ability, both in a static biofilm-forming system and under hydrodynamic conditions (Thornton et al., 2021; Thornton et al., 2023). In mice chronically infected with *P. aeruginosa*, RvD1 promoted phagocytosis of *P. aeruginosa* by lung macrophages and regulated the expression of Toll-like receptors, downstream genes, and microRNAs (MiR)-21 and 155, which reducesd inflammatory signals (Codagnone et al., 2018). Furthermore, in the context of co-infection with *Streptococcus* pneumoniae and influenza A virus, AT-RvD1 promoted faster clearance of pneumococcus from the lungs while reducing the severity of pneumonia by limiting excessive leukocyte chemotaxis from the infected bronchioles to the distal regions of the lungs (Wang et al., 2017). Taken together, these findings suggest that the prosolvency of SPMs represents a new host-directed therapeutic strategy to complement current antibiotic-centered approaches to fighting infections and lays the groundwork for further investigation of SPMs as an alternative to immunosuppressive therapies such as steroids.

SPMs have also been shown to exert effects in influenza virus infection. It has been reported that PD1 significantly inhibits influenza virus replication through an RNA export mechanism, which prevents fatal influenza virus infection and improves the survival of mice with severe influenza (Morita et al., 2013). 17-HDHA enhanced antibody-mediated anti-influenza virus immune response and is important for the development of new potential influenza vaccine adjuvants (Ramon et al., 2014).

### 6.4 Protective role of exogenous SPMs in the immunosuppression phase

Most studies have demonstrated the pro-resolving role of SPM in early sepsis, and more encouragingly there are also some studies suggesting that SPM may play a beneficial role in late sepsis. In the development of sepsis, the early manifestation is an inflammatory burst followed by a phase of immunosuppression characterized by lymphocyte apoptosis, monocyte/macrophage exhaustion, and increased migration of myeloid-derived suppressor cells (MDSCs), leading to host vulnerability to secondary infections (Padovani and Yin, 2024). The two-hit mouse model of CLP and secondary *P. aeruginosa* lung infection is a commonly used model of late polymicrobial sepsis. In studies using this mouse preclinical model, administration of RvD2 in the later stages of the CLP model (48 h postoperatively) demonstrated multiple beneficial effects:1) decreased blood and lung bacterial load; 2) increased phagocytosis by alveolar macrophages/monocytes 3) splenic T-cell counts were also increased 4) increased the number of non-inflammatory alveolar macrophages 5) increased mature neutrophils and MDSC accumulation in the spleen; 6) significantly decreased lung lavage levels of IL-23; and 7) decreased mortality (Walker et al., 2022; Sundarasivarao et al., 2022). These studies provide evidence that RvD2 promotes host defense mechanisms in sepsis and secondary lung infections and may have therapeutic value in the treatment of sepsis in the immunosuppressed phase.

## 7 Reflections on the results of human studies

Emerging clinical studies have reported associations between SPM levels and sepsis outcomes; however, it is critical to recognize that the analytical methodologies employed in many of these studies are now known to be inadequate for reliable SPM quantification, casting doubt on the validity of these reported associations. Multiple investigations report reduced systemic SPM concentrations (e.g., LXA4, MaR1) in septic patients compared to healthy controls, with lower levels correlating with increased disease severity, ARDS development, and mortality (Tsai et al., 2013; Jundi et al., 2021; Tejera et al., 2020). Importantly, these findings must be interpreted with extreme caution due to the methodological limitations discussed below. Parallel observations in COVID-19 indicate that SPM downregulation coincides with phagocyte dysfunction, while therapeutic interventions like dexamethasone may partially exert protection through SPM upregulation (Koenis et al., 2021). The clinical relevance of SPM receptors is further supported by findings that reduced GPR18 expression on neutrophils predicts poorer sepsis outcomes (Zhang L. et al., 2019). However, contradictory evidence exists: some studies observed elevated SPMs (RvE1, RvD5, 17R-PD1) alongside pro-inflammatory lipids in non-survivors, while others documented increased LXA4 and D-series resolvins in severe COVID-19 (Dalli et al., 2017; Archambault et al., 2021). These paradoxical reports likely arise primarily from methodological artifacts rather than biological phenomena, given the analytical shortcomings pervasive in this literature. Critically, it must be emphasized that no validated evidence exists for endogenous SPMs (e.g., RvD2, RvE1) in SALI samples, as earlier reports claiming their presence often misidentified analytical artifacts or failed LOD/LOQ validation (Schebb et al., 2025; O'Donnell et al., 2023).

These analytical limitations fundamentally undermine the reliability of purported clinical associations. Substantial analytical challenges complicate SPM quantification. Specifically, earlier clinical studies reporting SPM levels in sepsis patients frequently utilized methods now recognized as insufficiently rigorous (e.g., inadequate chromatographic resolution, failure to apply LOD/LOQ validation), rendering their correlative findings unsubstantiated. Concentrations in biological samples are frequently near detection limits (<10 pg/mL), with significant inter-study variability attributed to methodological differences in sample processing, storage, and LC-MS/MS analysis (Calder, 2020). Concerns regarding the validity of endogenous SPM measurements have been raised, particularly surrounding chromatographic data quality and inappropriate analytical practices in earlier literature. Consequently, rigorous adherence to internationally standardized protocols is essential for future biomarker studies.

Despite unresolved questions about endogenous biology, SPMs retain therapeutic promise. Clinical trials demonstrate that synthetic analogs (e.g., LXA4-stabilized mouthwash for periodontitis and RvE1 derivatives for dry eye disease) exhibit safety and efficacy in reducing inflammation (Hasturk et al., 2021; OphthalmologyWeb, 2024). However, the translational gap for life-threatening conditions like SALI partly stems from pharmacokinetic challenges: preclinical studies use highly variable SPM doses (0.1 ng/kg–5 μg/kg, Table 1) via intravenous/intraperitoneal routes, yet none measure systemic or lung tissue concentrations—a critical omission given SPMs’ rapid metabolism, poor tissue penetration, and enzymatic degradation. Recent mechanistic insights revealing SPMs require ∼100 nM concentrations for EP4 allosteric modulation (Alnouri et al., 2024)—orders of magnitude above endogenous levels—justify high-dose regimens but necessitate advanced delivery strategies. Promisingly, polycarbonate micelles prolong SPM release to 20 days while reducing oxidation (de Prinse et al., 2023), offering potential solutions to these bioavailability barriers. It is important to critically address the observation that clinical trials of SPM-based drugs have thus far primarily focused on less life-threatening conditions like periodontitis and dry eye disease, rather than severe systemic illnesses such as SALI. This strategic choice likely reflects several key considerations. Firstly, the safety threshold for initial human testing is understandably lower for localized, non-fatal inflammatory conditions. Topical or localized delivery (e.g., ocular administration for dry eye, oral rinse for periodontitis) offers practical advantages and minimizes systemic exposure concerns. Secondly, the pharmacokinetic limitations of natural SPMs pose a significant hurdle for systemic applications in critical illness. Natural SPMs are rapidly inactivated and cleared *in vivo*, demanding high doses for efficacy in preclinical sepsis models, which raises challenges related to drug formulation, stability, cost, and potential off-target effects at supraphysiological concentrations. These PK challenges necessitate the development of stable analogs or novel delivery systems before advancing into large-scale, resource-intensive trials for life-threatening conditions like sepsis. Furthermore, defining clinically relevant endpoints and patient populations for sepsis trials is inherently more complex than for conditions like dry eye. Nevertheless, the compelling preclinical evidence summarized herein, particularly their efficacy even in the immunosuppressive phase of sepsis and their potential to enhance host defense without broad immunosuppression, strongly motivates the imperative for future high-quality clinical trials directly evaluating SPM therapeutics in sepsis-associated lung injury. Such trials are essential to establish effective dosing paradigms and validate the translational potential of this resolution pharmacology approach. Notably, ω-3 fatty acid supplementation reduces sepsis mortality and ARDS incidence (Lei et al., 2023; Chen et al., 2018), though whether this reflects SPM generation remains contested (Souza et al., 2020; Sobrino et al., 2020; Marchand et al., 2023). Recent mechanistic insights reveal that supraphysiological SPM concentrations act via biased allosteric modulation of the EP4 receptor, converting anti-phagocytic signaling to pro-phagocytic activity—a critical pathway for inflammation resolution (Alnouri et al., 2024). This supports exploration of high-dose exogenous SPM administration to circumvent endogenous production limitations. Clinical trials directly evaluating SPM therapeutics in sepsis-associated lung injury are warranted to establish dosing paradigms and validate their translational potential.

**TABLE 1:** SPMs in septic lung injury.

SPMs	Model	Concentration of SPMs	Mechanisms of intervention	Effect/outcome	References
15-epi-LXA4	LPS intratracheal injection	0.7 mg/kg i.v	Pulmonary inflammation/oedema	Neutrophil↓, protein concentration↓, TNF-α, NO↓	Anesth Analg 2007; 104 (2): 369-77
			Oxidative stress	lipid peroxidation↓, HO-1↑, MDA↓	
		100–5000 ng/mouse i.v	Platelet-neutrophil interactions	Platelet activation↓, NPA↓	Blood 2014; 124 (17): 2625-34
	Live *E. coli* intraperitoneal injection/carrageenan plus MPO intratracheal injection	200 μg/kg i.v	Promote inflammation resolution	Mac-1, MPO, Akt, Mcl-1↓, phosphorylated Bad↓, neutrophil apoptosis↑, mitochondrial dysfunction↓	Am J Respir Crit Care Med 2009; 180 (4): 311-9
LXA4	CLP	① 0.1 mg/kg i.v ② 40 μg/kg i.p	Inflammation response/oxidative stress	PMN percentage↓, Macrophage↑, p38/MAPK, NF-κB↓, TNF-α, IL-6, MCP-1↓, MPO↓	① J Biol Regul Homeost Agents 2020; 34 (3): 807-814. ② Shock 2011; 36 (4): 410-6
		0.1 mg/kg i.v	Pulmonary edema/infection	AFC↑, W/D ratio of lung tissues↓, bacterial load↓	J Biol Regul Homeost Agents 2020; 34 (3): 807-814
	LPS intratracheal injection	① 100 μg/kg i.v ② 1 μg/mouse i.p ③ 2 μg/kg i.v	Pulmonary edema/inflammation response/oxidative stress	AFC, CFTR↑, neutrophil↓, IL-6, TNF-α↓, CAMP↑, Nrf2↑, ROS, mitochondrial oxidized status↓	① Free Radic Biol Med 2016; 93: 52-66. ② Respir Res 2019; 20 (1): 192. ③ Mediators Inflamm 2013; 2013: 862628
			AT II cells	Proliferation↑, PI3K/AKT, SMAD, caspase-3, apoptosis↓, E-cadherin↑, α-SMA, N-cadherin, vimentin↓, EMT↓	
	LPS intraperitoneal injection	20 μ g/kg i.v	Pulmonary edema	AP-1, miR-21↓, PTEN↑, AKT↓, ENaC-γ, AFC↑	Lab Invest 2015; 95 (11): 1258-68
		0.1 mg/kg,i.p	Pulmonary edema/inflammatory	AQP5, AFC↑, neutrophil↓, MPO↓, P-p38, P-JNK↓, TNF-α, IL-6↓	J Thorac Dis 2019; 11 (8): 3599-3608
R_V_D1	LPS Intratracheal injection	① 1 μg/kg or 5 μg/kg i.p ② 300 ng or 600 ng i.v ③ 100 ng/mouse i.v	Inflammation response/oxidative stress	Leukocytes, PMN, MPO↓, ICAM-1, VCAM-1, ELAM-1↓, NF-κB, MAPKs, PPARγ↓, IL-6, TNF-α, IL-1β↓, IL-10↑, CXCR4 (24 h) ↑, CXCR4 (72 h)↓, CXCL2, CXCL-12↓, HO-1, SOD↑, MDA↓	① Pulm Pharmacol Ther 2011; 24 (4): 434-41. ② Respir Res 2012; 13 (1): 110. ③ Chin Med J (Engl) 2014; 127 (5): 803-9. ④ J Surg Res 2014; 188 (1): 213-21. ⑤ Int Immunopharmacol 2019; 76: 105877. ⑥ Lab Invest 2013; 93 (9): 991-1000
		④ 3 μg/kg i.v ⑤ 0.1 μg/mouse i.p ⑥ 5 μg/kg i.v	Intercellular junction/apoptosis	Occludin, zona occludin-1↑, apoptosis↓	
	LPS intraperitoneal injection	5 μg/kg i.v	Pulmonary edema	ENaC↑, Na, K-ATP↑, ALX, cAMP, PI3K↑, AFC↑	J Immunol 2014; 192 (8): 3765-77
	CLP	10 ng/g i.v	Inflammation response/oxidative stress	STAT3, NF-κB, MAPKs↓, Sirtuin 1↑, IL-6, TNF-α, IL-1β↓, MPO↓	Inflammation 2018; 41 (5): 1762-1771
R_V_D2	Secondary lung infection after CLP	① 100 ng/mouse i.v ② 100 ng/mouse i.v	Pulmonary infection/cytokine	Pulmonary bacterial load↓, non-inflammatory macrophages↑, splenic T-cells↑, TLR-2↑, IL-23↓	① Prostaglandins Other Lipid Mediat 2022; 159: 106617. ② Front Immunol. 2022; 13:1011944
			Bacterial clearance	Splenic neutrophils and MDSCs↑	
R_V_E1	Human alveolar epithelial cells	50 nM	Stress and apoptosis	HSP60, p38ɑ, PON2, COX-2, SIRT2, SOD2, p21↓, TRAIL R2↓	Lipids 2019; 54 (1): 53-65
			Mitochondrial function/inflammations	Maximal respiratory capacity per cell↑, FIS1↓, MFN1↑, IL-6, IL-8↓	
AT-RvD1	Live *E. coli* or *P. aeruginosa* intratracheal injection	100 ng/mouse i.v	Inflammatory cells	macrophage phagocytosis and efferocytosis ↑, M2 macrophage↑	Mucosal Immunol. 2016 September; 9 (5):1278-87
			Bacterial clearance	anti-microbial peptide lipocalin 2 ↑	
PD1	LPS intratracheal injection	200 ng/mouse i.v	Inflammatory cells	Neutrophil counts↓, myeloperoxidase↓, apoptosis↑	Chin Med J (Engl) 2014; 127 (5): 810-4
			Pulmonary edema	Protein in BALF, W/D ratio of lung tissues↓	
PDX	LPS intratracheal injection	① 1 ng, 10 ng or 100 ng i.v ② 0.1ng/mouse i.v	Inflammation cells	Neutrophil-platelet activate↓, macrophage, neutrophil recruit↓	① Chin Med J (Engl) 2018; 131 (10): 1167-1173. ② J Cell Mol Med 2020; 24 (18): 10604-10614
			Inflammatory factor	TNF-α, MCP-1, MIP-2, MMP9↓	
	CLP	300 ng i.p	Inflammatory cells/phagocytosis	Arg1, Ym1, PPAR-γ↑, M2 macrophage↑, bacterial load↓	Sci Rep 2017; 7 (1): 99
		500 ng or 1000 ng i.p	Inflammatory response	PPARγ↑, NF-κB↓, IL-1β, IL-6, TNF-ɑ, MCP-1↓, IL-10↑	Immunol Res 2020; 68 (5): 280-288
			Lung permeability and edema	Protein, leukocyte, neutrophil in BALF↓,W/D ratio↓	
	LPS intraperitoneal injection	5 μg/kg i.v	Alveolar fluid clearance	Na, K-ATPase, sodium channel↑, Nedd4-2↓, P-Akt↑, cAMP↑, AFC↑	Exp Mol Med 2018; 50 (4): 1-13
PCTR1	LPS intraperitoneal injection	1 μg/kg i.v	Alveolar fluid clearance	Na, K-ATPase, sodium channel↑, LYVE-1↑, Nedd4-2↓, P-Akt↑, cAMP↑, AFC↑	J Cell Physiol 2020; 235 (12): 9510-9523
		100 ng/mouse i.p	Pulmonary vascular permeability/edema	HS and SDC-1 in lungs↑, HPA↓, EXT-1↑, W/D ratio, EBD↓	Respir Res 2021; 22 (1): 193
			Inflammation response	SIRT1↑, NF-κB↓, TNF-α, IL-6 and IL-1β↓	
PCTR1	LPS intraperitoneal injection	100 ng or 200 ng i.v	ferroptosis	Fe2+, PTGS2 and ROS↓, GSH and GPX4↑	J Transl Med. 2023 April 30; 21 (1):293
MaR1	LPS Intratracheal injection	① 0.1 ng or 1 ng i.v ② 1 ng i.v ③ 10 ng i.v ④ 10ng, 50 ng or 100 ng/mouse i.p	Inflammatory cells/cytokines	Neutrophil↓, M2 macrophage↑, MAPK/PI3K↓, P-selection, IL-6, TNF-α, IL-1β↓	① Br J Pharmacol 2014; 171 (14): 3539-50. ② Shock 2015; 44 (4): 371-80. ③ J Surg Res 2020; 256: 584-594. ④ Lab Invest 2018; 98 (6): 715-733
			Apoptosis/oxidative stress	Bcl-2, Mcl-1↓, neutrophil apoptosis↑, ICAM-1, MPO↓	
	LPS intraperitoneal injection	200 ng/kg i.v	Pulmonary edema	Na, K-ATPase, ENaC↑, PI3k/Akt↑, Nedd4-2↓, AFC↑	Lab Invest 2017; 97 (5): 543-554
	CLP	① 10ng, 50 ng or 100 ng/mouse i.p ② 1 ng i.v	Inflammatory response/bacterial clearance	Neutrophils↓, NF-κB↓, TNF-α, IL-6 and IL-1β↓, bacterial load and LPS↓	① Lab Invest 2018; 98 (6): 715-733. ② Mediators Inflamm. 2016; 2016:3798465
			Mitochondria	NOX↓, CAT and SOD↑, ROS and mtO2↓ ALX, cAMP and mtDNA↑	
MCTR1	LPS intraperitoneal injection	100 ng/mouse i.p	Inflammatory response	SIRT1↑, NF-κB↓, TNF-α, IL-6 and IL-1β↓	J Cell Physiol 2020; 235 (10): 7283-7294
			Endothelial cells	HS, syndecan-1 and HPA↓	
MCTR3	LPS enterocoelia injection	2 ng/g i.p	Inflammation response/oxidative stress	TNF-α↓, MCP-1 ↓, IL-1β↓, MDA ↓, SOD ↑	Int Immunopharmacol. 2021 January:90:107142
			Mitochondria	Apoptosis ↓, PINK1 pathway proteins ↓	

Abbreviations: 15-epi- lipoxin A4 (ATL); lipopolysaccharide (LPS); cecal ligation and puncture (CLP); mitogen-activated protein kinases (MAPKs); nuclear factor-κB (NF-κB); alveolar fluid clearance (AFC); tumor necrosis factor-α (TNF-α), polymorphonuclear neutrophils (PMN); myeloperoxidase (MPO); extracellular signal–regulated kinase (ERK); NF-E2-related factor 2 (Nrf2); alveolar epithelial type II, cells (AT II, cells); epithelial-mesenchymal transition (EMT); alveolar epithelial sodium channel (ENaC); phosphatase and tensin homolog (PTEN); cystic fibrosis transmembrane conductance regulator (CFTR); Aquaporin (AQP); malondialdehyde (MDA); superoxide dismutase (SOD); signal transducer and activator of transcription 3 (STAT3); Protectin conjugates in tissue regeneration 1 (PCTR1); f intercellular adhesion molecule (ICAM); NADPH, oxidase (NOX); catalases (CAT); macrophage inflammatory protein 2 (MIP-2); monocyte chemoattractant protein-1 (MCP-1); matrix metalloproteinase 9 (MMP9); small mother against decapentaplegic (SMAD); bronchoalveolar lavage fluid (BALF); wet/dry (W/D); neutrophil-platelet aggregates (NPA); heparan sulfate (HS); syndecan-1 (SDC-1); heparanase (HPA); exostosin-1 (EXT-1); PTEN-induced putative kinase 1 (PINK1); prostaglandin-endoperoxide synthase 2 (PTGS2).

## 8 Conclusion

While current supportive therapies (anti-infectives, fluid resuscitation, mechanical ventilation) have improved survival in SALI, they fail to address the underlying immune dysregulation driving the pathology. This review synthesizes compelling preclinical evidence demonstrating that exogenously administered SPMs represent a promising therapeutic strategy targeting these fundamental mechanisms. As detailed in Table 1, we comprehensively summarize the multi-faceted protective actions of diverse SPMs in SALI: ① Epithelial & Endothelial Protection: SPMs restore AFC by enhancing ENaC, Na,K-ATPase, CFTR, and AQP5 function, reducing pulmonary edema. They attenuate AECs apoptosis and inhibit EMT. Concurrently, SPMs maintain endothelial glycocalyx homeostasis by downregulating HPA and upregulated EXT-1 expression, preserving vascular barrier integrity. ② Mitigation of Oxidative Stress & Mitochondrial Dysfunction: SPMs reduce ROS overproduction, enhance antioxidant defenses (e.g., via Nrf2 activation), restore inflammation-impaired mitochondrial respiratory capacity, and correct mitochondrial fission/fusion imbalances. ③ Pro-Resolving Immunomodulation: Critically, and distinct from broad immunosuppression, SPMs actively promote resolution by: Reducing neutrophil infiltration and NETosis, enhancing macrophage efferocytosis and phagocytic clearance. Promoting macrophage polarization towards an M2 phenotype. Dampening pro-inflammatory cytokine/chemokine production (e.g., TNF-α, IL-1β, IL-6, MIP-2, MCP-1) and modulating key signaling pathways (NF-κB, MAPK, STAT, PPARγ). Modulating microbial virulence and enhancing antibiotic efficacy. Significantly, exerting protective effects even during the immunosuppressive phase of sepsis.

It is essential to note significant controversies regarding the physiological relevance of endogenous SPM generation and their proposed cognate GPCR signaling pathways. Critically, and as emphasized throughout this review, robust detection of physiologically relevant concentrations of specific SPMs (e.g., RvD2, RvE1) in biological matrices, particularly SALI samples, remains unvalidated. Earlier studies frequently misattributed analytical artifacts (e.g., solvent peaks, contaminants) to SPMs due to methodological flaws, including failures to apply standard LOD or LOQ criteria (O'Donnell et al., 2023). Consequently, internationally standardized LC-MS/MS protocols have now been established to ensure rigorous identification and quantification (Schebb et al., 2025). To date, no study adhering to these validated standards has definitively demonstrated the presence of endogenous SPMs in human SALI samples. Recent mechanistic insights reveal that SPMs (e.g., protectins, maresins, D-series resolvins) function as biased positive allosteric modulators of the EP4 receptor at supraphysiological concentrations. This EP4-dependent action converts anti-phagocytic signaling to pro-phagocytic activity-a central mechanism underpinning the therapeutic efficacy of exogenously administered SPMs observed in preclinical models (Alnouri et al., 2024). The requirement for supraphysiological concentrations (EC_50_ ∼100 nM) to activate this pathway highlights the pharmacokinetic challenges of natural SPMs, including rapid clearance and undetectable lung tissue levels in existing studies. Future formulations must therefore prioritize sustained-release systems (e.g., oxidation-protective micelles) to maintain therapeutic exposure.

The robust preclinical evidence summarized herein strongly supports the therapeutic potential of exogenously delivered SPMs or their stable analogs for SALI. Translating this promise requires: ① High-quality clinical trials evaluating SPMs/analogs in SALI patients, building on positive safety/efficacy signals in other inflammatory conditions (e.g., LXA4 analog in periodontitis (Hasturk et al., 2021); RvE1 analog in dry eye OphthalmologyWeb (2024). ② Exploration of optimized delivery strategies, informed by the EP4 allosteric modulation mechanism, to overcome potential drug-likeness challenges (e.g., high-dose regimens, stable analog development, targeted delivery systems). ③ Continued mechanistic refinement to fully elucidate optimal dosing paradigms and molecular targets, integrated with pharmacokinetic assessments of lung tissue exposure; and ④ Development of delivery platforms that counteract rapid clearance, such as polymeric micelles for sustained SPM release (de Prinse et al., 2023).

In conclusion, while questions regarding endogenous SPM physiology persist, exogenous SPMs represent a novel resolution pharmacology-based approach to actively restore immune homeostasis in SALI. Focused efforts on clinical translation hold significant potential to open new therapeutic avenues for this devastating condition.
